# Modeling Native EHEC Outer Membrane Vesicles by Creating Synthetic Surrogates

**DOI:** 10.3390/microorganisms8050673

**Published:** 2020-05-06

**Authors:** Alexander Kehl, Ronja Kuhn, Johanna Detzner, Daniel Steil, Johannes Müthing, Helge Karch, Alexander Mellmann

**Affiliations:** 1 Institute of Hygiene, University of Münster, 48149 Münster, Germany; ronja.kuhn@ukmuenster.de (R.K.); johanna.detzner@ukmuenster.de (J.D.); daniel.steil87@gmail.com (D.S.); jm@uni-muenster.de (J.M.); helge.karch@ukmuenster.de (H.K.); 2 National Consulting Laboratory for Hemolytic Uremic Syndrome (HUS), University of Münster, 48149 Münster, Germany

**Keywords:** EHEC, liposome, OMV, Stx, toxin, vesicle

## Abstract

Enterohemorrhagic *Escherichia coli* (EHEC) is a zoonotic pathogen responsible for life-threating diseases such as hemolytic uremic syndrome. While its major virulence factor, the Shiga toxin (Stx), is known to exert its cytotoxic effect on various endothelial and epithelial cells when in its free, soluble form, Stx was also recently found to be associated with EHEC outer membrane vesicles (OMVs). However, depending on the strain background, other toxins can also be associated with native OMVs (nOMVs), and nOMVs are also made up of immunomodulatory agents such as lipopolysaccharides and flagellin. Thus, it is difficult to determine to which extent a single virulence factor in nOMVs, such as Stx, contributes to the molecular pathogenesis of EHEC. To reduce this complexity, we successfully developed a protocol for the preparation of synthetic OMVs (sOMVs) with a defined lipid composition resembling the *E. coli* outer membrane and loaded with specific proteins, i.e., bovine serum albumin (BSA) as a proxy for functional Stx2a. Using BSA for parameter evaluation, we found that (1) functional sOMVs can be prepared at room temperature instead of potentially detrimental higher temperatures (e.g., 45 °C), (2) a 1:10 ratio of protein to lipid, i.e., 100 µg protein with 1 mg of lipid mixture, yields homogenously sized sOMVs, and (3) long-term storage for up to one year at 4 °C is possible without losing structural integrity. Accordingly, we reproducibly generated Stx2a-loaded sOMVs with an average diameter of 132.4 ± 9.6 nm that preserve Stx2a’s injuring activity, as determined by cytotoxicity assays with Vero cells. Overall, we successfully created sOMVs and loaded them with an EHEC toxin, which opens the door for future studies on the degree of virulence associated with individual toxins from EHEC and other bacterial pathogens.

## 1. Introduction

Enterohemorrhagic *Escherichia coli* (EHEC), a highly human-pathogenic subgroup of Shiga-toxigenic *Escherichia coli* (STEC), can cause gastrointestinal infections accompanied by bloody diarrhea, hemorrhagic colitis, or life-threatening hemolytic uremic syndrome (HUS) [1,2], whereby HUS involves thrombocytopenia, microangiopathic hemolytic anemia, and acute renal failure and potentially also neurological sequelae [3]. The cardinal virulence factor of EHEC is the Shiga toxin (Stx), belonging to the AB_5_ toxin family, where the B subunit binds to host cells and the A subunit is catalytically active [4,5]. During pathogenesis, Stx’s main targets are renal glomerular and brain microvascular endothelial cells [6,7], but other cell types, such as intestinal epithelial cells, renal epithelial cells or even erythropoietic cells, are also subject to Stx intoxication [8,9,10]. Generally, Stx exerts its *N*-glycosidase activity onto ribosomes, ultimately leading to the inhibition of protein biosynthesis and finally cell death [4,5], but Stx also targets other subcellular structures such as DNA [11].

Even though many studies have analyzed virulence factors, including Stx, in their free, soluble form, recently studies have found that virulence factors are also carried on the pathogenic bacteria’s outer membrane vesicles (OMVs) [12], which are nanostructures released during growth by all gram-negative bacteria [13]. This has also been confirmed for EHEC, as Stx was found to be associated with OMVs [14,15]. Moreover, since EHEC can exhibit rather diverse genomic backgrounds [16], different strains can encode highly varying sets of virulence factors, and many of these factors have likewise been identified to be OMV-associated, including the EHEC-hemolysin (EHEC-Hly), the *Shigella* enterotoxin 1 (ShET1), and the cytolethal distending toxin V (CdtV) [17,18,19]. However, although studies have investigated the impact of the free, soluble toxins on cellular barriers as well as how the OMV-associated forms are trafficked, it is still unknown how much each virulence factor associated with OMVs contributes to EHEC pathogenesis. Further, previous studies have generally analyzed native OMVs (nOMVs), in which the associated virulence factors are considered as an ensemble rather than individually. An additional confounding issue is that the surfaces of nOMVs also exhibit lipopolysaccharides (LPS) and, in motile strains, flagellin, both of which strongly modulate the secretion of proinflammatory cytokines, an important factor in HUS [20,21,22,23].

Therefore, to downscale the natural complexity of nOMVs in order to study only selected components, here we developed an approach to create an OMV-like environment without LPS and/or flagellin, allowing us to analyze associated virulence factors in defined compositions. For this, we first adapted existing protocols for the preparation of liposomes/vesicles using a distinct lipid composition and then loaded them with desired proteins; the resulting products are termed synthetic OMVs (sOMVs). After establishing various parameters using bovine serum albumin (BSA) as a proxy, we successfully generated sOMVs loaded with Stx2a, the Stx subtype most often correlated with HUS [24]. Importantly, these Stx2a-loaded sOMVs were able to damage Vero cells, indicating that sOMV-associated Stx2a retains its cytotoxicity even when not in its free, soluble form.

## 2. Materials and Methods

### 2.1. Preparation of Protein-Loaded Vesicles

Commercial lipids, employed for the preparation of liposomes, were all obtained from Avanti Polar Lipids (Alabaster, AL, USA) and comprised 1-palmitoyl-2-oleoyl-*sn*-glycero-3-phosphoethanolamine (POPE), 1-palmitoyl-2-oleoyl-*sn*-glycero-3-phospho-(1′-*rac*-glycerol) (POPG), and 1′,3′-bis[1,2-dioleoyl-*sn*-glycero-3-phospho]-*sn*-glycerol (cardiolipin, CL). A volume of 1 mL of the lipid mixture (1 mg total lipid, if not otherwise stated) in chloroform/methanol (2/1, v/v) was dried in a rotary evaporator at 45 °C. The resulting lipid film was dissolved in 1 mL of water and incubated 2× for 10 min at 45 °C in a water bath sonicator (Sonorex, Bandelin, Berlin, Germany). Liposomes were prepared with a mini-extruder (Avanti Polar Lipids) by extruding the suspension through a polycarbonate membrane with a 100 nm pore size (Whatman Nucleopore Track-Etched Membranes, GE Healthcare, Maidstone, UK) at 45 °C [25]. A volume of 1 mL with (if not otherwise stated) either 1 mg fatty acid-free bovine serum albumin (BSA, fraction V, receptor grade, lyophilized; Serva Electrophoresis, Heidelberg, Germany) or 100 µg Stx2a was added in overall 0.1× PBS, and the mixture was lyophilized in a Beta 1-8 LSCplus instrument (Christ, Osterode am Harz, Germany).

Stx2a was affinity-purified from the Stx2a-containing supernatant of *E. coli* strain 03-0616 (O111:H^-^) as previously described [26]. Briefly, concentrated Stx2a-containing bacterial culture supernatants were incubated with globotriaose-spiked magnetic beads, and bound Stx2a was subsequently eluted. After comprehensive structural characterization by mass spectrometry, functionality was verified by real-time interaction analyses and cytotoxicity assays (see also below) [8,26,27]. Density gradient-purified nOMVs from *E. coli* strain HUSEC029 (O70:H8) were likewise prepared as previously described [17]. Briefly, OMVs were sedimented from bacterial culture supernatants by ultracentrifugation, and the pellet was applied onto an OptiPrep density gradient and then ultracentrifuged again. Stx2a-positive fractions were finally concentrated by another ultracentrifugation step.

The protein-lipid precipitate was first rehydrated with 0.1 mL water for 30 min and then incubated with 0.9 mL PBS for another 30 min, both at room temperature (RT). The suspension was again extruded as above and finally centrifuged thrice at 40,000× *g* and 4 °C for 60 min in an Optima XPN-80 ultracentrifuge (Beckman Coulter, Krefeld, Germany); the supernatant (sn) was replaced with fresh PBS between centrifugation runs.

The particle size distribution of the resulting sOMVs was determined by dynamic light scattering (DLS) measurements with a Zetasizer Nano ZS instrument (Malvern Instruments, Malvern, UK) at a fixed 173° backscatter angle (non-invasive backscatter) with 10 s runs at a number automatically adjusted by the instrument [26]. The average diameter (Z-average size) of OMVs and the index of the particle size distribution (polydispersity index, PDI) were analyzed with the Zetasizer software (version 7.13). If sOMVs were not sufficiently homogenous after centrifugation, an additional extrusion step was performed. Protein concentrations were determined using a bicinchoninic acid assay (Pierce BCA Protein Assay Kit, #23225, ThermoFisher Scientific, Dreieich, Germany) following the supplier’s instructions [28], in which sOMVs were solubilized by Triton X-100. The encapsulation efficiency (EE) was determined as follows:

EE (%) = (protein_sOMVs_/(protein_sOMVs_ + protein_sn_)) × 100 (1)

### 2.2. Cell Culture and Cytotoxicity Assay

Vero-B4 cells (German Collection of Microorganisms and Cell Cultures, DSMZ; Braunschweig, Germany; no. ACC 33) were routinely cultured in a humidified atmosphere at 37 °C with 5% CO_2_ in DMEM (Lonza, Cologne, Germany) supplemented with 10% fetal calf serum (FCS; PAA, Pasching, Austria) and 2 mM ultraglutamine (Lonza).

Stx2a-mediated cellular damage was assessed with the crystal violet assay as previously described [9,29]. In short, 5 × 10^3^ cells per well were seeded onto 96-well plates (Corning, Corning, NY, USA) in 100 µL cell culture medium. After 24 h, 100 μL of 10× dilutions of free, soluble Stx2a, sOMVs, or nOMVs in cell culture medium (starting with 50 μg/mL) or 100 μL of the medium, as a control, were applied onto subconfluent cells and incubated for another 72 h. The subsequent fixation of cells and measurement of cell viability, performed on an OPTIMA instrument (BMG LABTECH, Offenburg, Germany), followed in detail the previously described protocol [9,29]. Results represent the mean ± standard deviation of three biological replicates (*n* = 3) performed in triplicate determinations and are displayed as percentage values of untreated control cells. Statistical analyses were performed with SigmaPlot 13 (Systat Software, Erkrath, Germany).

## 3. Results

**Figure 1:**
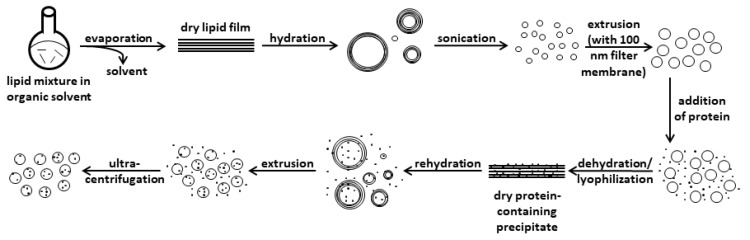
Workflow for synthetic outer membrane vesicle (sOMV) preparation. Depicted is a combined process by which vesicles are homogenized through an extrusion step without proteins, and then the protein is added and the vesicles are dehydrated and rehydrated. The protein-loaded products are again homogenized by extrusion, and excess protein is removed by ultracentrifugation. Adapted from Walde and Ichikawa [30] and modified.

The rationale for creating sOMVs was to be able to test the virulence of individual EHEC virulence factors incorporated into vesicles. Several approaches exist to achieve this goal [30]. We decided to apply a combination of different techniques, which is summarized in Figure 1. In this procedure, we followed a protocol established in our lab for steps including the preparation of vesicles up until the addition of protein [26]. For the following steps, we used another protocol as the basis and modified it accordingly [31].

### 3.1. Developing a Protocol for the Preparation of sOMVs

We started by generating a dry lipid film of a mixture of 1 mg of total lipid consisting of 60% POPE, 30% POPG, and 10% CL (Figure 1). As we wanted to exclude LPS but still sought to mimic an OMV-like-environment, this mixture was meant to resemble the inner leaflet of the outer membrane of *E. coli*, which consists mainly of PE and lower amounts of PG and CL, containing mostly saturated or unsaturated C16 or C18 fatty acids [32,33]. This and the following steps up until the addition of the intended protein were executed at a temperature of 45 °C (Figure 1), as vesicle preparation should occur at an ambient temperature 5–10 °C above the *T*_m_, the main lamellar chain-melting phase transition temperature [30]. This is mandatory to avoid the crystalline state and preserve the fluid state of the lipids. As the *T*_m_ is not readily deducible from complex lipid mixtures, the temperature of 45 °C, which has also been applied previously [26], was empirically identified to be suitable for this specific lipid mixture. This dry lipid film was then hydrated with water, which is obligatory when preparing protein-loaded vesicles to achieve proper buffer concentrations (see below), and then it was sonicated to disrupt multi-layered structures (Figure 1). Finally, this mixture was extruded with a 100 nm filter membrane to result in a homogenous vesicle suspension, as shown in Figure S1A.

For the second half of the procedure, the intended protein (1 mg) was added to the vesicle suspension from the first half of the procedure, thus resulting in a 1:1 protein-lipid ratio (Figure 1). Subsequently, the protein-lipid mixture was dehydrated by lyophilization and then rehydrated to ultimately achieve a proper buffer concentration. Thus, we added the protein at this step in a buffer concentration 10× less concentrated than usual, since the first step of rehydration involved adding a volume of water 10× less than the initial concentration to avoid disrupting the structural integrity of vesicles via higher ionic strength [30]. Then, the resulting multi-layered structures were disrupted by extrusion (again with a 100 nm filter membrane), and finally, excess protein in the supernatant was removed by low-speed multi-step ultracentrifugation (Figure 1). In the case that the particle size distribution of the resulting sOMV mixture showed aggregates when measured with DLS, an additional extrusion step was carried out.

### 3.2. Defining Parameters for sOMV Preparation Using BSA as a Proxy

**Figure 2:**
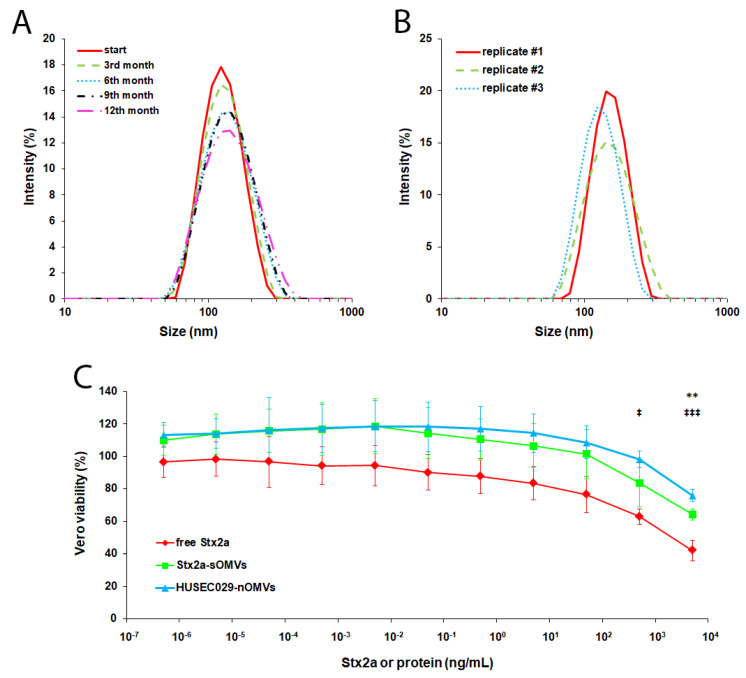
Long-term storage of sOMVs and properties of Stx2a-loaded sOMVs. Depicted in (**A**,**B**) is the size distribution as measured by dynamic light scattering (DLS). (**A**) Bovine serum albumin (BSA)-loaded sOMVs were stored at 4 °C and analyzed at the indicated time-points for structural integrity. A sample representative of three biological replicates is shown over time. (**B**) Size distribution of three biological replicates of Stx2a-loaded sOMVs (also stored at 4 °C) indicated by differently colored lines. (**C**) Cytotoxicity of free Stx2a, Stx2a-loaded sOMVs, and Stx2a-containing HUSEC029 native outer membrane vesicles (nOMVs) toward Vero cells, as determined by the crystal violet assay. The indicated concentrations refer to Stx2a for measurements with free Stx2a and Stx2a-loaded sOMVs or total protein for nOMVs of HUSEC029, respectively. Depicted is the mean ± standard deviation in relation to an untreated control of three biological replicates (*n* = 3) each performed in triplicates. Statistical analyses were performed by ANOVA with Bonferroni correction comparing free Stx2a with Stx2a-sOMVs (*) or with HUSEC029-nOMVs (‡), respectively, and significances are indicated as follows: */‡, *p* < 0.05; **/‡‡, *p* < 0.01; ***/‡‡‡, *p* < 0.001 (comparing Stx2a-sOMVs with HUSEC029-nOMVs yielded no significant differences).

Instead of loading the vesicles with valuable toxins right away, we used BSA as a proxy to define critical parameters. First, as a preparation temperature of 45 °C would be potentially detrimental for the eventual toxins we planned to use, we tested whether RT might also be suitable for the preparation of sOMVs. As shown in Figure S1B, our approach is not only suitable to prepare a homogenous population of sOMVs in general, but it is also able to be performed at RT, as the Z-average sizes of 132.1 nm and 141.6 nm at 45 °C and RT, respectively, are in a similar range. Next, as various protocols often suggest different lipid or protein amounts or ratios [31,34], we probed varying ratios and found that using 1 mg of lipid yields a slightly more homogenous sOMV preparation than 10 mg (Figure S1C). Besides, a 1:10 protein-lipid ratio results in sOMVs similar to a 1:1 ratio, thus being advantageous for toxin expenditure. Finally, as vendors of consumables for vesicle preparation recommend storing such preparations at 4 °C for no longer than one week, we analyzed whether sOMVs could be frozen for storage. However, neither conventionally freezing sOMVs in the freezer (−20 °C) nor potentially more structure-sustaining deep-freezing them with liquid nitrogen (−196 °C) preserved the sOMVs’ vesicular structures, as shown in Figure S1D. Thus, because we had to store prepared sOMVs at 4 °C for more than one week if they were not used immediately, to test the limits of sOMV shelf stability we examined whether long-term storage at 4 °C was possible without losing the structural integrity of the sOMVs; in fact, as shown in Figure 2A, we found that sOMVs can be stored for up to one year at 4 °C without a significant loss of integrity.

### 3.3. Preparing and Functionally Evaluating sOMVs Harboring Stx2a

After we defined the proper parameters for the preparation of sOMVs using BSA, we next loaded the sOMVs with an actual EHEC toxin, Stx2a. As with BSA, we were able to reproducibly generate Stx2a-loaded sOMVs, as shown in Figure 2B with a similar size distribution (Z-average diameter of 132.4 ± 9.6 nm with a PDI of 0.107 ± 0.042) and a protein concentration of 33.3 ± 30.2 µg/mL and an EE of 54.7% ± 23.6%. However, simply forming the sOMVs does not guarantee the entrapped protein displays biological activity. Thus, we applied sOMVs in cytotoxicity assays onto Vero cells, the gold standard cell line for this purpose [35,36]. To exclude any potential unspecific effects from the lipids used in the sOMV preparation procedure or the carrying of cargo by sOMVs in general, we first applied empty sOMVs and BSA-loaded sOMVs onto Vero cells as depicted in Figure S2. Indeed, we did not observe any detrimental effect except for the highest concentration, which, however, is most likely due to the high amount of PBS, in which sOMVs were ultimately reconstituted during preparation, applied onto cells before being diluted for lower concentrations. Next, we performed cytotoxicity assays with Stx2a-loaded sOMVs and, for comparison, also with free, soluble Stx2a and nOMVs from HUSEC029, an EHEC strain with Stx2a but no other major EHEC toxin [16]. As shown in Figure 2C, Stx2a-loaded sOMVs indeed exerted a cytotoxic effect, where the dose dependence strongly resembled that of both free Stx2a and HUSEC029 nOMVs. However, the cytotoxic effect of the Stx2a-loaded sOMVs was, overall, not as pronounced as that of free Stx2a, though the difference was only statistically significant with the highest concentration. The dose dependence was instead more comparable, showing no significant differences, to that of HUSEC029 nOMVs, which itself was significantly different to free Stx2a only at higher concentrations. Taken together, we not only successfully established a protocol for the generation of toxin-harboring sOMVs, but the resulting vesicles were also able to preserve the toxin’s harmful activity.

## 4. Discussion

Here, we describe an approach to incorporate EHEC toxins, using Stx2a as a proof of principle, into sOMVs in order to reduce the natural complexity of EHEC nOMVs down to only one virulence factor. Different approaches to generate Stx-associated vesicles have been attempted before. In one series of studies, Stx1a and Stx2a were coupled to vesicles via glutaraldehyde for the analysis of their immunogenic potential, which detoxified the toxins [37,38,39]. Similarly, another study examined the immunogenicity of functionalized vesicles, though these were loaded only with the non-toxic B subunit of Stx2a [40]. The B subunit grafted onto vesicles was also used in a series of studies that aimed to quantify adsorption of such spherical structures to cells [41,42,43]. In a different study, whole-cell antigen preparations of *E. coli* O157:H7 were entrapped in vesicles, also for studying immunization [44]. However, not all of these approaches attempted to incorporate intact holotoxins and, more importantly, none of them aimed to preserve the toxin’s cytotoxic activity to analyze its function. We now present a method that allows for using toxin-harboring vesicles in phenotypic assays, as our approach successfully sustained the toxin’s detrimental activity.

Of note, several parameters determined here for Stx2a-loaded sOMVs are similar to those of nOMVs deriving from EHEC. First, an average size of 132.4 nm is similar in size to the nOMVs of EHEC O157 strains, which ranged from 92.1 to 180.8 nm [19]. Further, a protein concentration of 33.3 µg/mL in sOMVs (here directly representing the Stx2a concentration) relatively closely resembles the Stx2a concentrations detected in nOMVs from EHEC O104:H4, O157:H7, and O157:H^-^ with 58 µg/mL, 51 µg/mL, and 50 µg/mL, respectively [18,19]. Hence, our approach represents ideal conditions to analyze the effect of this EHEC toxin in its OMV-associated form with physiologically relevant prerequisites. Although it remains to be determined why the Stx2a-sOMVs overall do not induce cytotoxicity as strongly as the free, soluble Stx2a, it might indicate that not all of the Stx molecules survived the procedure, even though the majority must have, as demonstrated by the pronounced cytotoxic effect at higher toxin concentrations. Interestingly, the effect was noticeably similar to that of HUSEC029 nOMVs, again underlining the suitability of sOMVs as a surrogate for nOMVs. The slightly lower cytotoxicity of HUSEC029 nOMVs at higher concentrations might have occurred for two reasons: First, the indicated concentrations correspond to total protein; thus, the amount of Stx2a within the HUSEC029 nOMVs might have been significantly lower than in the Stx2a-sOMVs (and free Stx2a). Second, the complex molecule mixture in nOMVs might have contained disturbing factors that could have potentially hampered the full activity of the toxin.

Collectively, in this study we provide an approach for studying single virulence determinants, especially toxins, in an OMV-like environment. In the future, our approach can be applied to scrutinize other toxins known to be OMV-associated, i.e., the EHEC-Hly, ShET1, and CdtV [17,18,19]. Eventually, such investigations could even be expanded to include varying combinations of these toxins to analyze possible synergistic effects and to unravel the contributions of different EHEC virulence factors to pathogenesis.

## Supplementary Materials

The following are available online at https://www.mdpi.com/2076-2607/8/5/673/s1, Figure S1: Parameters evaluated for sOMV preparation, Figure S2: Effect on cell viability of lipids used in sOMV preparation and cargo in general.

## Abbreviations

CdtV	cytolethal distending toxin V
EHEC	enterohemorrhagic *Escherichia coli*
EHEC-Hly	EHEC hemolysin
nOMVs	native OMVs
OMVs	outer membrane vesicles
ShET1	*Shigella* enterotoxin 1
sOMVs	synthetic OMVs
STEC	Shiga-toxigenic *Escherichia coli*
Stx	Shiga toxin
