# Supplementary material for: Modeling Native EHEC Outer Membrane Vesicles by Creating Synthetic Surrogates
Source: Microorganisms. 2020 May 6;8(5):673. doi: 10.3390/microorganisms8050673 (PMC7284840; doi:10.3390/microorganisms8050673)
Supplement: Supplementary file 1 [file microorganisms-08-00673-s001.pdf]

Supplementary Materials

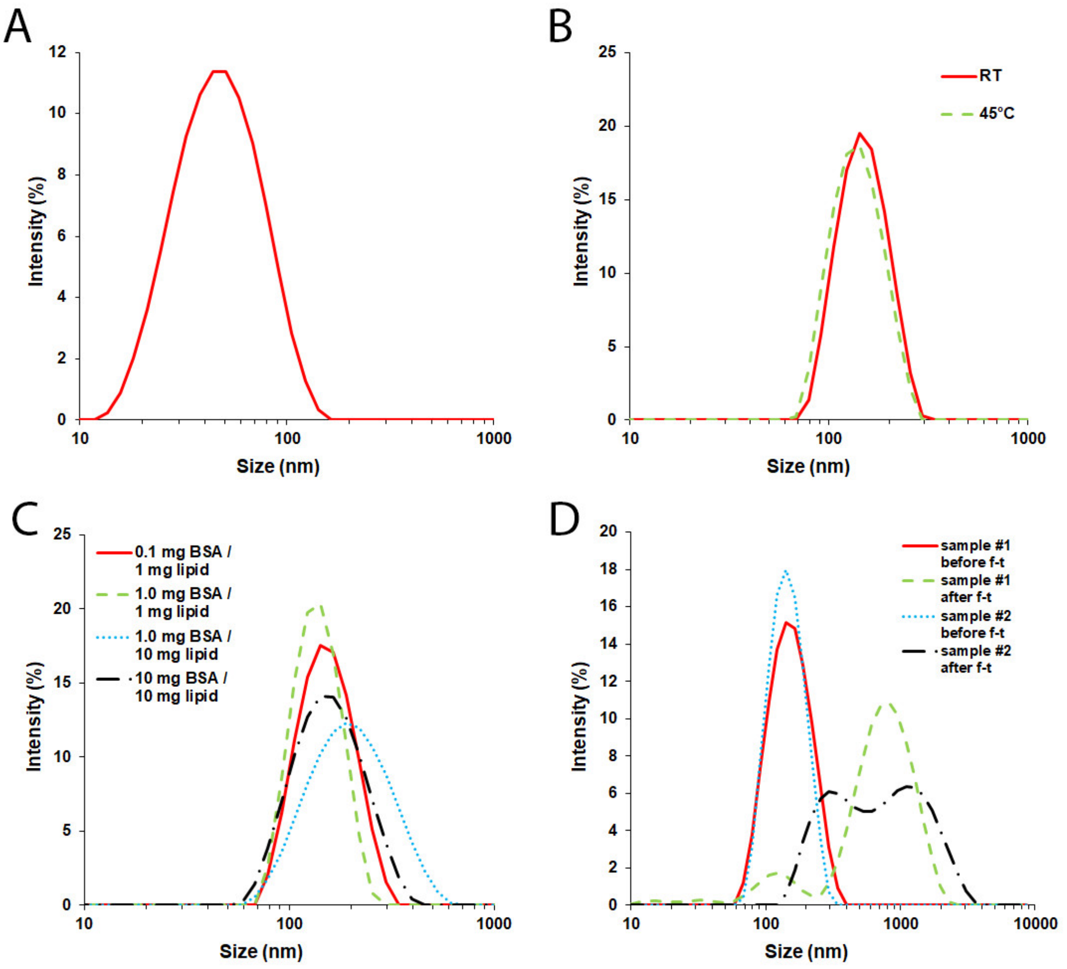

Figure S1. Parameters evaluated for sOMV preparation. Depicted is the size distribution of liposomes (**A**) and sOMVs (**B**-**D**) as measured by DLS. (**A**) POPG/POPE/CL liposomes after extrusion and before the addition of protein. (**B**) BSA-loaded sOMVs after ultracentrifugation with the complete preparation procedure performed at 45 °C or changed to RT upon addition of protein. (**C**) BSA-loaded sOMV preparations with different ratios of protein and lipid, as indicated. (**D**) BSA-loaded sOMV preparations initially stored at 4 °C before and after freezing with subsequent thawing (freeze-thawing, f-t) either conventionally frozen in the freezer at -20 °C (#1) or deep-frozen in liquid nitrogen at -196 °C (#2).

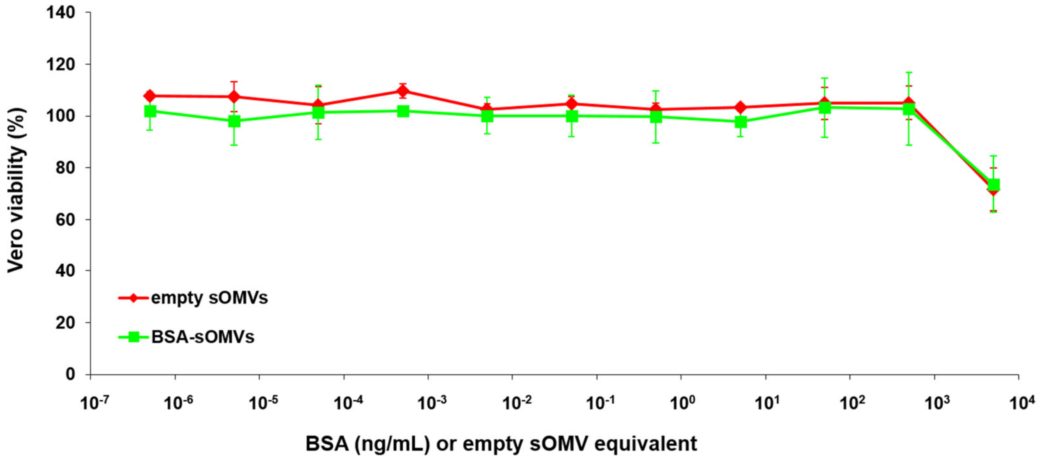

Figure S2. Effect on cell viability of lipids used in sOMV preparation and cargo in general. Cytotoxicity of empty sOMVs without cargo and BSA-loaded sOMVs toward Vero cells, as determined by the crystal violet assay. For empty sOMVs, an amount equivalent to the one of BSA-loaded sOMVs was applied. Depicted is the mean ± standard deviation in relation to an untreated control of three biological replicates (n = 3) each performed in triplicates.
